# Assessing Telemedicine Implementation Potential in High‐Referral Specialties: A Cross‐Sectional Study in Iran

**DOI:** 10.1002/hsr2.73021

**Published:** 2026-08-26

**Authors:** Reyhane Izadi, Abdosaleh Jafari, Zahra Zare, Mohammad Amin Bahrami

**Affiliations:** ^1^ Department of Health Care Management, School of Management and Information Sciences Shiraz University of Medical Sciences Shiraz Iran; ^2^ Health Human Resources Research Center, School of Health Management and Information Sciences Shiraz University of Medical Sciences Shiraz Iran

**Keywords:** medical specialties, Shiraz University of Medical Sciences, telehealth, telemedicine, telemedicine implementation

## Abstract

**Background and Aims:**

Telemedicine is more than just an advanced technology; it represents a fundamental transformation in the delivery of healthcare services, facilitating seamless communication between patients and specialists through innovative means that are unrestricted by geographical limitations. This study aimed to identify medical specialties with the highest referral rates and assess their readiness for telemedicine implementation within the population covered by Shiraz University of Medical Sciences.

**Methods:**

The study was conducted in two stages: first, identifying medical specialties with the highest patient referral frequencies, and second, assessing the potential for telemedicine implementation in these key specialties. Specialties were selected using a referral frequency threshold defined as one standard deviation above the mean number of total referrals (mean + 1 SD). To evaluate telemedicine implementation potential, four key indicators were assessed—specialists' willingness to use telemedicine, financing, organizational capacity, and practical equipment—through surveys of experts with relevant knowledge and experience in the field. A composite index was then calculated to rank the specialties, and a sensitivity analysis using alternative weighting schemes was performed to examine the stability of the rankings.

**Results:**

The analysis revealed that a total of 21,328 patient referrals were recorded within the studied population during the year under review, with 70.8% (15,099) of these referrals pertaining to six key medical specialties. An assessment of telemedicine implementation potential across the six specialties showed that general surgery demonstrated the highest score (1.55), followed by cardiology (1.47), general internal medicine (1.40), orthopedics (1.38), neurosurgery (1.36), and pediatrics (1.06), representing a descending gradient from the most to the least favorable status.

**Conclusion:**

The assessment of telemedicine implementation potential in key medical specialties has identified the most suitable fields for the expansion of this technology. By analyzing the critical factors influencing telemedicine adoption, this study provides actionable insights for policymakers to design targeted implementation strategies, such as sustainable financing models, enhancement of technological infrastructure, supportive regulatory frameworks, and removal of specialty‐specific barriers. These recommendations can guide the advancement of remote healthcare services in high‐demand specialties.

## 1 Introduction

The rapid evolution of technology, the emergence of novel innovations, and intensifying competition have positioned the adoption of advanced technologies at the core of business enterprises and governmental strategies. Among its profound influences on various aspects of human society, information technology has played a pivotal role in shaping healthcare by facilitating the emergence of information‐driven societies. At present, healthcare processes are fundamentally reliant on technological advancements, as their management, transmission, elimination of geographical barriers, acceleration of diagnosis, and enhancement of treatment methods are indispensable considerations. These two overarching themes have led to the establishment of a specialized branch within medical sciences, known as telemedicine technology [1].

The concept of telemedicine was first defined in 1971 when Bird described it as “the practice of medicine without direct physician‐patient confrontation, enabled by an interactive audio‐video communication system” [2]. The World Health Organization (WHO) offers a widely accepted definition, describing telemedicine as “the delivery of healthcare services across distances, utilizing information and communication technologies to exchange valid data for diagnosis, treatment, disease prevention, research, evaluation, and the continuous education of healthcare providers, all in service of improving individual and community health” [3].

Telemedicine is revolutionizing healthcare delivery, enabling automatic remote health monitoring for patients who cannot be physically present due to distance or medical conditions. By enhancing medical service provision, telemedicine strengthens healthcare systems and facilitates early disease detection through teleconsultation, particularly for conditions with subtle or overlooked symptoms. Moreover, it accelerates the exchange of expertise among medical professionals, fostering collaboration and innovation [2, 4, 5].

Despite its transformative potential, telemedicine implementation faces challenges. Barriers to its effective integration stem not only from patient‐related factors but also from healthcare professionals and the structural organization of medical institutions [2, 6, 7]. The advancement of systems facilitating remote diagnosis and patient monitoring is fundamentally driven by progress in information technology, telecommunications, and telematics. The integration of telemedicine into clinical practice has revealed challenges including legal, financial, technological, and awareness‐related barriers [2].

In addition to these general considerations, understanding patient referral patterns is essential for evaluating telemedicine potential. According to the WHO, referral is the process through which a healthcare provider at a particular level of the health system—who lacks the necessary resources, such as equipment, medications, or clinical expertise, to manage a patient's condition—requests support from another facility with greater or different capabilities at the same or a higher level of care to assist with or assume responsibility for the patient's management [8].

In this study, high‐referral specialties are defined as medical disciplines that receive the highest volume of patient referrals across hospitals affiliated with Shiraz University of Medical Sciences (SUMS) as well as other medical universities in the region. These referrals occur between facilities at different levels of care through the Monitoring Center for Medical Care (MCMC) system. Such specialties bear the greatest inter‐facility referral burden, often due to limited specialist availability, complex diagnostic requirements, and high service demand. Identifying referral patterns in these fields is crucial for evaluating the potential for telemedicine implementation, as remote care solutions can reduce avoidable transfers, enhance equitable access to specialist expertise, and improve coordination throughout the referral system [9].

Given this substantial referral burden, the institutional context of SUMS further underscores the importance of evaluating telemedicine readiness in these specialties. As the largest academic and tertiary referral hub in southern Iran, SUMS oversees a wide network of county hospitals, specialty centers, and primary care units. Its extensive catchment area generates significant referral volumes through the MCMC system, particularly in specialties experiencing chronic service bottlenecks. This institutional context makes SUMS a highly relevant setting for assessing telemedicine readiness in high‐referral specialties, where the potential impact of digital service delivery is most substantial.

Recent studies have demonstrated the feasibility and effectiveness of telemedicine implementation across diverse healthcare settings. For example, a large‐scale prehospital telemedicine system in Germany significantly improved guideline adherence and patient safety while reducing emergency physician interventions and helicopter‐based medical missions following implementation [10]. Similarly, an assisted telemedicine model in India integrating electronic health records and clinical decision support systems improved healthcare accessibility and achieved measurable reductions in blood sugar and blood pressure levels among patients with chronic diseases [11]. In addition, a cross‐sectional survey conducted in Germany highlighted that successful telemedicine adoption depends not only on technological readiness, but also on infrastructure, regulatory frameworks, and public perception at the health‐system level [12].

Despite these promising outcomes, the sustainability and scalability of telemedicine services vary considerably across medical specialties and healthcare environments. Evidence suggests that telemedicine adoption is strongly influenced by specialty‐specific requirements as well as the socio‐economic context of healthcare systems. Studies from middle‐income countries have identified persistent barriers, including limited digital infrastructure, resource constraints, and disparities in digital awareness between healthcare sectors [13]. In telepsychiatry, effective implementation is often challenged by shortages of trained professionals and difficulties in task‐shifting approaches [14]. Likewise, tele oncology faces complex regulatory and clinical delivery challenges [15], whereas tele dermatology implementation is frequently hindered by technological limitations related to image standardization and workflow integration [16]. Collectively, these findings indicate that although telemedicine has substantial potential to improve healthcare delivery, successful implementation requires specialty‐oriented and context‐sensitive strategies rather than a uniform approach across all medical fields.

Given the variability in telemedicine adoption across healthcare systems and medical specialties, there remains a need for comprehensive insights into the feasibility of its implementation in different clinical contexts. Although previous studies have demonstrated the potential benefits of telemedicine, limited research has systematically explored expert perspectives on the feasibility and barriers of telemedicine implementation across multiple specialties within referral healthcare settings. In the context of SUMS Sciences as a major referral healthcare center, the present study aimed to evaluate the feasibility and perceived barriers of telemedicine implementation in high‐referral specialties through the perspectives of clinical experts and decision‐makers. The findings of this study may provide evidence‐based guidance for policymakers, healthcare managers, and healthcare professionals seeking to develop specialty‐sensitive telemedicine strategies and optimize implementation in high‐demand clinical settings.

## 2 Methods

### 2.1 Study Design

This is a cross‐sectional study aimed at determining the potential for implementing telemedicine in specialties with high referral frequency at Shiraz University of Medical Sciences. The methodology of this study was adopted from the “Needs‐based Planning Framework for Telemedicine Services” guideline [17]; and was conducted in two main phases. In the first phase, key medical specialties were identified, and in the second phase, the implementation potential of each specialty was assessed based on four criteria, after which these key specialties were ranked for implementation. The recruitment period for this study in order to distribute and collect forms started from 2025‐02‐15 and ended on 2025‐04‐15.

### 2.2 Phases of the Research Method

#### 2.2.1 Phase 1: Identifying the List of Key Medical Specialties With the Highest Patient Referral Rates

In this phase, high‐demand medical specialties, which due to a shortage of specialists have led to patient referrals outside the geographical area of their residence, were identified for the provision of services through telemedicine. Analysis of referral data can help make strategic decisions about implementing telemedicine for specialties with the highest referral volumes [17]. Using data from the Monitoring Center for Medical Care (MCMC) system, which is considered the most comprehensive source of patient referral information within the target community (areas covered by SUMS), this list of specialties was determined.

#### 2.2.2 Phase 2: Evaluation of Telemedicine Implementation Potential in Key Specialties

The primary objective of this phase was to assess the status of key indicators for telemedicine implementation, differentiated by the specialties required within the studied community, in order to determine the implementation potential of telemedicine for each specialty. The primary outcome was the telemedicine implementation potential index, representing the overall implementation potential score for each specialty. The indicators examined in this phase included specialists' willingness to use telemedicine, financing, organizational capacity, and practical equipment. These four indicators were considered the secondary outcomes and were assessed individually to calculate the primary outcome. Ultimately, an overall implementation potential score was calculated for each specialty based on these four criteria. The criteria examined are as follows: First, clinicians' willingness assesses physicians' overall readiness and inclination to adopt telemedicine services. Second, practical equipment focuses on the technical and infrastructural aspects, including the availability and support for medical equipment, internet connectivity, physical space, and related resources necessary for effective telemedicine delivery. Third, organizational capacity evaluates an institution's ability to manage change and the impact of such changes on healthcare delivery processes. Fourth, financing examines the adequacy, provision, and sustainability of financial resources required to support telemedicine implementation. These four domains collectively served as secondary outcome measures for calculating the primary outcome.

### 2.3 Sampling and Data Collection

Two data sources were used in this study: the referral patient information system and an expert survey. The Referral Patient Information System at Shiraz University of Medical Sciences (Monitoring Center for Medical Care, MCMC) was used to identify medical specialties with the highest referral frequencies, while the expert survey was employed to assess the status of the four key indicators of implementation potential.

A census approach was applied to review all patient referrals within the regions covered by Shiraz University of Medical Sciences. For expert selection, a purposive snowball sampling method was employed. Experts were required to be employed within the regions under Shiraz University of Medical Sciences' coverage, provide written informed consent, and possess relevant knowledge or experience. Additional inclusion criteria included a medical specialty in one of the six most frequently referred fields, at least 2 years of professional experience in the university's educational or treatment centers, administrative or programmatic/committee experience related to financing, organizational capacity, or telemedicine implementation—or practical experience with telemedicine in their specialty—and introduction by at least one qualified specialist during the snowball sampling process. Exclusion criteria comprised incomplete survey responses or withdrawal from the study.

The expert panel, composed of senior administrators from Shiraz University of Medical Sciences and researchers, was convened to identify key individuals in priority medical specialties based on patient referral volume. Subsequently, surveys were conducted online and via telephone following prior coordination. The survey continued using snowball sampling. Recruitment continued until no additional eligible experts meeting the inclusion criteria were identified, indicating practical saturation within this highly specialized expert population. Given that telemedicine development within Shiraz University of Medical Sciences is still limited, the number of specialists who simultaneously possessed relevant clinical expertise and sufficient knowledge of telemedicine implementation, financing mechanisms, and organizational requirements was inherently small; therefore, the final sample of experts represents a substantial proportion of the available qualified population.

### 2.4 Data Extraction

Two forms were used for data extraction in this study. The first form collected information from the referral patient information system, specifying the reason for referral (specialty type) and referral frequency. The second form was for conducting an expert survey; it initially included a question for written consent from respondents. It then included questions related to criteria that could potentially influence the implementation of telemedicine, including the willingness of specialties to use telemedicine, financing, organizational capacity, and practical equipment. The data collection tool was developed based on the “Needs‐based planning for telemedicine services” guideline, complemented by a review of relevant literature, expert panel input, and the use of ChatGPT (OpenAI) solely as an auxiliary tool to support the initial translation and refinement of wording. All AI‑assisted suggestions were reviewed and adjusted by the research team to ensure conceptual accuracy and contextual appropriateness. Its validity was assessed in terms of face and content validity by clinical, managerial, and research experts. Face validity was examined using four criteria—comprehensibility, clarity, non‐ambiguity, and reduced risk of misinterpretation—rated on a 10‐point scale. The overall mean score was 8.38 ± 0.27, indicating strong face validity. Following expert recommendations, three items were revised.

Content validity was evaluated using the Item‐level Content Validity Index (I‐CVI), Scale‐level Content Validity Index (S‐CVI), and modified kappa statistics. Each item was rated on a four‐point relevance scale. The I‐CVI values ranged from 0.72 to 1.00, leading to revisions in two items scoring below 0.79. The S‐CVI was calculated as 0.83, confirming acceptable content validity at the scale level. The mean modified kappa value was 0.76, indicating substantial agreement among experts. Overall, after applying the necessary modifications, the instrument demonstrated an acceptable level of validity for accurately measuring the intended constructs. The full questionnaire used in the expert survey has been provided as Supporting file to ensure methodological transparency and enable future replication of the study. The reliability of the questionnaire was assessed using Cronbach's alpha. Values above 0.7 indicate acceptable internal consistency. In this study, the overall scale showed good reliability (α = 0.81).

### 2.5 Analysis

Quantitative data extracted from the information system were categorized based on the frequency of patient referral reasons (specialty type), and a list of specialties with the highest referral rates was identified using the area under the normal distribution curve (mean + standard deviation). The mean +standard deviation was employed as a threshold to identify specialties exhibiting substantially higher referral volumes, providing a straightforward yet robust approach consistent with established practices for detecting values exceeding the typical range in normally distributed data. As evidence suggests, specialties with a high volume of patient referrals serve as a key criterion for determining the necessity of telemedicine in the relevant fields [17].

The four criteria— willingness of specialties to use telemedicine, financing, organizational capacity, and practical equipment—were assessed using 16 questions and a Likert scale. The Likert scale responses were assigned standard numerical values: for “Financing”, “Organizational capacity,” and “Practical equipment,” scores ranged from 1 (worst) to 3 (best), while for “Willingness to use telemedicine,” scores ranged from 1 (Very Low) to 5 (Very High). The three organizational criteria—financial provision, organizational capacity, and practical equipment—exhibit limited, clearly distinguishable variation, justifying a three‐level scale. In contrast, willingness to use telemedicine reflects individual attitudes with greater nuance, warranting a five‐level scale. This tailored approach ensures each domain is measured with appropriate sensitivity, enhancing the precision and validity of the composite index.

The score for each criterion was calculated using an unweighted mean, assigning equal weight to all items within that criterion. Subsequently, each criterion was categorized according to predefined classification intervals. For the first three criteria, classifications were: 1–1.66 (poor), 1.67–2.33 (moderate), and 2.34–3 (good). For “Willingness to use telemedicine,” classifications were: 1–1.8 (very low), 1.81–2.6 (low), 2.61–3.4 (moderate), 3.41–4.2 (high), and 4.21–5 (very high). In the next step, equal weight was assigned to all four criteria. Their scores were then aggregated through simple summation, and based on the total score (i.e., the sum of the four criteria), a composite index (primary outcome: implementation potential) was constructed. Specialties were ranked according to this composite index. It should be noted that assigning equal weight to the four criteria is consistent with the “Need‐Based Framework for Telemedicine Service Planning” [13], which served as the methodological foundation of this study. Furthermore, to facilitate comparison across the four criteria and the resulting composite index, the overall score of each criterion as well as the composite index were normalized to a 0–1 scale for each of the six required specialties. This normalization enabled clearer and more consistent comparative analysis (Normalized Score = (Raw Score−Minimum)/(Maximum−Minimum)). First, the raw scores of the four criteria were normalized to a 0–1 scale, and then the composite index was calculated by summing these normalized scores.

The resulting classifications indicate each specialty's readiness for telemedicine implementation. Specialties with higher aggregated scores are expected to adopt telemedicine more effectively, indicating sufficient organizational capacity, adequate financing, and practical equipment. Conversely, specialties with lower scores may face greater barriers and require targeted support or resources to ensure successful telemedicine integration.

### 2.6 Sensitivity Analysis

To ensure the robustness and reliability of the final rankings, a sensitivity analysis was performed by varying the weights assigned to the four criteria. Two distinct scenarios were compared [1]: a baseline scenario based on the study's initial guidelines, where all four criteria were assigned equal weights (0.25 each), and [2] an expert‐driven scenario, where weights were redistributed based on the expert panel's consensus. In the latter, “Specialists' Willingness” was assigned a higher weight of 0.4, while the remaining three criteria (Practical equipment, Organizational Capacity, and Financing) were weighted at 0.2 each. This approach assessed the sensitivity of the final prioritization to changes in expert judgment and weight allocation. The higher weight assigned to “Specialists' Willingness” reflected the expert panel's consensus. Although this study did not systematically investigate the rationale for this weighting, experts generally perceived clinicians' willingness as the primary prerequisite for successful telemedicine implementation, while financing, organizational capacity, and practical equipment were considered enabling conditions that support, rather than determine, implementation.

### 2.7 Ethical Considerations

This study was approved by the National Ethics Committee in Iranian Biomedical Research, which was registered with the code of ethics approval “IR.SUMS.NUMIMG.REC.1402.044” on July 15, 2023, and all the methods adopted in the present study were in accordance with the relevant instructions and regulations. Participation was voluntary informed consent was obtained prior to survey completion. To maintain confidentiality, no personal identifiers were collected. All responses were anonymized at the point of data entry, and no attempts were made to re‐identify respondents.

All survey responses were reviewed for completeness. In cases of missing or incomplete data, participants were contacted for clarification, and any unretrievable data were excluded from the analyses to ensure accuracy and validity.

## 3 Results

### 3.1 Key Specialties for Delivery Via Telemedicine

The selection of key specialties for telemedicine delivery was determined based on the frequency of referrals. In total, six specialties were identified: neurosurgery, cardiology, general surgery, general internal medicine, orthopedics, and pediatrics (Table 1). The total number of patient referrals in the target population in the year under review was 21,328, of which 15,099 (70.8%) were related to these six key specialties.

**Table 1:** Selected specialties for telemedicine delivery.

No.	Medical specialties	Number of patient referrals
1	Neurosurgery	3488
2	Cardiology	3407
3	General surgery	2281
4	General internal medicine	2076
5	Orthopedics	2058
6	Pediatrics	1789
*	Total	15099

Table 2 presents the demographic and professional characteristics of the 31 expert participants involved in the survey. Experts were recruited using purposive sampling, and participation was arranged through prior coordination with eligible specialists. All 31 experts who were invited agreed to participate and completed the survey, resulting in a 100% response rate. These experts were senior medical specialists with influential roles in clinical decision‐making across the selected specialties. As shown in the table, 61.3% of participants were male, and the average age was 38.4 ± 3.2 years. All specialists had at least 2 years of service within the regions covered by Shiraz University of Medical Sciences. Their distribution across the six high‐referral specialties included 7 experts in orthopedics, 4 in pediatrics, 3 in general surgery, 6 in general internal medicine, 5 in cardiology, and 6 in neurosurgery.

**Table 2:** Demographic and professional characteristics of the expert participants.

Characteristic	*N* (%) or mean ± SD
**Total experts**	31
**Age, years**	38.4 ± 3.2
**Gender**	
Male	19 (61.3%)
Female	12 (38.7%)
**Medical specialty**	
Orthopedics	7 (22.5%)
Pediatrics	4 (12.9%)
General surgery	3 (9.7%)
General internal medicine	6 (19.4%)
Cardiology	5 (16.1%)
Neurosurgery	6 (19.4%)
**Work experience**	≥ 2 years at Shiraz University of Medical Sciences
**Geographical area of service**	Areas covered by Shiraz University of Medical Sciences

### 3.2 Implementation Potential of Telemedicine in Key Specialties Based on Four Indicators

#### 3.2.1 Specialists' Willingness to Use Telemedicine/Criterion 1

The average score for this criterion was 2.84 out of 5, indicating a moderate level of acceptance. Cardiologists exhibited the highest inclination toward delivering services via telemedicine, whereas pediatric specialists showed the least enthusiasm for adopting telemedicine‐based service delivery (Table 3).

**Table 3:** Specialists' willingness to use telemedicine.

No.	Medical specialties	Average score (score out of 5)
1	Neurosurgery	2.80
2	Cardiology	3.20
3	General surgery	2.66
4	General internal medicine	3.16
5	Orthopedics	3.00
6	Pediatrics	2.25
*	Total	2.84

#### 3.2.2 Financing/Criterion 2

The average score for the financing criterion was 1.28 out of 3, placing it in the poor category. Budget adequacy showed the lowest score and fell within the weak range, whereas the other two dimensions were within the moderate range. Neurosurgery received the highest overall score, while pediatrics had the lowest among the key specialties (Table 4).

**Table 4:** Financing status in key specialties.

No.	Medical specialties	Dimensions of financing			Average score (score out of 3)
		Financial support impact	Budget adequacy	Dedicated budget allocation	
1	Orthopedics	1.42	1.00	1.28	1.23
2	Pediatrics	1.50	1.00	1.00	1.16
3	General surgery	1.66	1.00	1.00	1.22
4	General internal medicine	1.33	1.00	1.66	1.33
5	Cardiology	1.60	1.00	1.40	1.33
6	Neurosurgery	1.33	1.33	1.66	1.44
—	Total	1.47	1.05	1.33	1.28

#### 3.2.3 Organizational Capacity/Criterion 3

The average overall score for organizational capacity to modify care delivery processes using telemedicine was 2.06 out of 3, placing it in the moderate range. Additionally, both organizational support and adequacy of training and resources for change were rated moderate. Moreover, experts determined that telemedicine can have a highly positive impact on three key aspects of daily workflow, with the greatest effect observed in service recipient satisfaction, followed by efficiency and service quality. The highest average score for organizational capacity to modify care delivery processes using telemedicine was observed in the following specialties, in descending order: general surgery, orthopedics, cardiology, general internal medicine, neurosurgery, and pediatrics. This criterion was rated moderate across all specialties (Table 5).

**Table 5:** Status of organizational capacity in key specialties.

Medical specialties	Dimensions of organizational capacity					Average score (score out of 3)
	Impact of telemedicine on daily workflow			Adequacy of training and resources	Organizational support	
	Service recipient satisfaction	Quality	Efficiency			
Orthopedics	2.85	2.71	2.71	1.14	1.14	2.11
Pediatrics	2.50	1.50	3.00	1.00	1.50	1.90
General surgery	3.00	3.00	2.66	1.33	1.33	2.26
General internal medicine	2.66	2.50	2.83	1.00	1.33	2.06
Cardiology	3.00	2.20	3.00	1.20	1.00	2.08
Neurosurgery	2.50	2.16	2.16	1.66	1.50	1.99
Total	2.75	2.34	2.72	1.22	1.30	2.06

#### 3.2.4 Practical Equipment/Criterion 4

The score for the practical equipment criterion was 1.45 out of 3, placing it in the poor range. Both the overall average score and the scores of its dimensions were in the poor range, with the exception of the general surgery department, which achieved a mean score within the moderate category. Additionally, the results indicate that the practical equipment scores varied across specialties, ranked from highest to lowest as follows: general surgery, pediatrics, cardiology, orthopedics, neurosurgery, and general internal medicine (Table 6).

**Table 6:** Status of practical equipment in key specialties.

Medical specialties	Dimensions of practical equipment					Average score (score out of 3)
	IT support services	Reliable internet connectivity	Dedicated space for telemedicine sessions	Support, maintenance, and upgrades of telemedicine equipment	Availability of telemedicine equipment	
Orthopedics	1.42	1.57	1.14	1.28	1.71	1.42
Pediatrics	1.50	1.50	1.00	1.25	2.00	1.45
General surgery	2.00	1.66	2.00	1.66	1.66	1.79
General internal medicine	1.33	1.50	1.00	1.33	1.50	1.33
Cardiology	1.40	1.60	1.40	1.20	1.60	1.44
Neurosurgery	1.33	1.16	1.16	1.66	1.66	1.39
Total	1.49	1.49	1.28	1.39	1.68	1.45

#### 3.2.5 Telemedicine Implementation Potential Index

The telemedicine implementation potential index (primary outcome), based on the four key criteria (secondary outcomes), revealed that the following specialties ranked from first to sixth place in order: general surgery, cardiology, general internal medicine, orthopedics, neurosurgery, and pediatrics (Table 7).

**Table 7:** Telemedicine implementation potential index in key specialties.

Secondary outcomes									Primary outcome	Rank
Medical specialties	Financing		Organizational capacity		Practical equipment		Specialists' willingness		Total score	
	Score out of 3	Normalized score	Score out of 3	Normalized score	Score out of 3	Normalized score	Score out of 5	Normalized score	Normalized total score	
Orthopedics	1.23	0.115	2.11	0.555	1.42	0.210	3.00	0.500	1.380	4
Pediatrics	1.16	0.080	1.90	0.450	1.45	0.225	2.25	0.312	1.067	6
General surgery	1.22	0.110	2.26	0.630	1.79	0.395	2.66	0.415	1.550	1
General internal medicine	1.33	0.165	2.06	0.530	1.33	0.165	3.16	0.540	1.400	3
Cardiology	1.33	0.165	2.08	0.540	1.44	0.220	3.20	0.550	1.475	2
Neurosurgery	1.44	0.220	1.99	0.495	1.39	0.195	2.80	0.450	1.360	5

**Figure 1:**
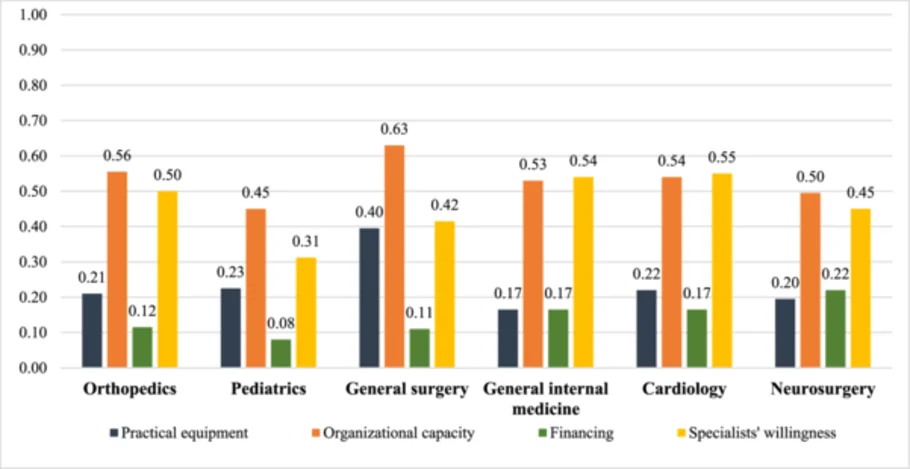
Comparative telemedicine readiness scores across six high‐referral specialties based on key dimensions.

Figure 1 compares normalized telemedicine readiness scores across six high‐referral specialties in four dimensions: practical equipment, organizational capacity, financing, and specialists' willingness. To facilitate rapid comparison and highlight key strengths and limitations, all scores are presented on a standardized 0–1 scale. The data illustrate that organizational capacity is highest in general surgery, whereas specialists' willingness peaks in cardiology. Conversely, financing consistently receives the lowest scores across all specialties, indicating a cross‐cutting constraint on readiness.

#### 3.2.6 Sensitivity Analysis

The results of the sensitivity analysis indicated that the prioritization model is highly stable. As shown in Table 8, comparing the equal‐weight scenario with the expert‐weighted scenario revealed only a minor shift in the top positions. Specifically, the first and second ranks (Cardiology and General Surgery) swapped places with a negligible score differential of approximately 0.01. All other medical specialties maintained their original rankings across both scenarios. This minimal variation confirms that the proposed composite index is resilient to weight adjustments and that the identified priorities are statistically sound.

**Table 8:** Comparison of Specialty Rankings under Different Weighting Scenarios.

Medical specialties	Equal weights (Baseline)	Rank	Expert weights (Scenario 2)	Rank	Rank change
Cardiology	0.368	2	0.405	1	+1
General surgery	0.387	1	0.393	2	−1
General internal medicine	0.350	3	0.388	3	0
Orthopedics	0.345	4	0.376	4	0
Neurosurgery	0.340	5	0.362	5	0
Pediatrics	0.266	6	0.275	6	0

## 4 Discussion

Although this study used a needs‐based telemedicine planning framework [17], its conceptual basis aligns with established telemedicine readiness models. Its multidimensional approach—covering financial, operational, user willingness, and organizational capacity—resembles the structure of the Telemedicine Hat Model, which evaluates technical, organizational, and institutional aspects. The study's emphasis on expert engagement and specialty‐specific assessment reflects stakeholder and community readiness dimensions in the Telemedicine Community Readiness Model [18]. Additionally, prioritizing medical specialties based on referral volume parallels the pre‐assessment phase of the Model for Assessment of Telemedicine [19]. The following paragraph outlines the study's objectives and key findings.

This study assessed the implementation potential of telemedicine in high‐referral medical specialties within the population covered by Shiraz University of Medical Sciences. Overall, an evaluation of telemedicine implementation potential—based on expert surveys in these six key specialties—revealed the following ranking, from first to sixth place: general surgery, cardiology, general internal medicine, orthopedics, neurosurgery, and pediatrics. The results of the sensitivity analysis showed that when equal and alternative weights were applied to the criteria, only a minor shift was observed between the top two ranks. The findings of this study are consistent with telemedicine adoption patterns observed during the COVID‑19 pandemic as reported by Patel et al [20]., with higher implementation potential in cardiology and internal medicine paralleling their broader use of telemedicine during the pandemic, while lower readiness in orthopedic specialty corresponds to their more limited adoption. Further details on the status of these key specialties, based on the criteria examined in this study, are discussed below, supported by similar evidence. Although some studies have explored telemedicine adoption, few have compared implementation readiness across medical specialties. Accordingly, this discussion draws on the most relevant available evidence to place the current findings in context.

Specialists' willingness to adopt telemedicine varied across medical fields. Cardiology showed the highest readiness, likely due to the compatibility of its workflows with remote care, including follow‐up visits for chronic heart failure, hypertension monitoring, medication adjustments, and review of diagnostic data such as ECG and echocardiography. Cardiologists' frequent reliance on digital diagnostics and longitudinal patient monitoring further facilitates seamless telemedicine integration. In contrast, pediatrics scored lowest, as care depends heavily on direct physical examination, behavioral observation, and interaction with caregivers. Younger children's limited ability to communicate symptoms, combined with concerns about diagnostic uncertainty, medicolegal responsibility, and variability in caregivers' digital literacy, reduces pediatricians' confidence in remote care. Although no study directly compares specialties, several works provide insight into adoption factors. The Technology Acceptance Model (TAM) identifies perceived usefulness and ease of use as key determinants, while institutional support, regulatory frameworks, and technological infrastructure also play major roles [21, 22, 23, 24]. Empirical evidence supports these findings: 77.7% of cardiac professionals expressed willingness to expand telemedicine [25], whereas pediatric adoption is hindered by provider biases and misalignment with technological workflows, highlighting the need to refine processes to address clinician and caregiver concerns [26].

Financing was a moderate constraint on telemedicine implementation, with an average score of 1.28 out of 3. Budget adequacy scored lowest, while dedicated budgets and financial support were moderate. Pediatrics received the lowest score, reflecting its reliance on frequent in‐person assessments, routine vaccinations, and preventive care, which offer limited economic return for telemedicine investments. In contrast, specialties such as cardiology, neurosurgery, and internal medicine involve chronic disease monitoring, diagnostic data review, and follow‐up visits, which can be efficiently delivered remotely, optimizing staff and equipment use and reducing patient transfers. Although few studies directly compare financing across specialties, prior research highlights general challenges: hospitals face limited reimbursement for telehealth, low patient volume, in‐person care preferences, and insufficient bandwidth, all of which constrain financial sustainability [27]. Moreover, many countries lack value‐based telemedicine billing, particularly for preventive services in chronic disease management [28]. Aligning telemedicine reimbursements with in‐person equivalents is therefore essential to support sustainable adoption [29, 30, 31].

Organizational capacity to implement telemedicine was generally good, with an average score of 2.06 out of 3. While organizational support and adequacy of training were moderate, experts noted that telemedicine can positively impact service recipients' satisfaction, efficiency, and quality of care. Specialties such as general surgery, orthopedics, and cardiology scored highest, benefiting from structured protocols, defined care pathways, multidisciplinary teamwork, and prior experience with technological adaptation. In contrast, pediatrics faces challenges due to direct child‐caregiver interactions, complex workflows, ethical and legal considerations, and limited prior telemedicine experience, which constrain organizational readiness. Organizational capacity is a critical determinant of successful telemedicine adoption, enabling managers to assess readiness and guide change processes [32]. Fragmented change management and insufficient planning have been linked to slower adoption rates [33]. Evidence suggests that adapting workflows, investing in staff training, and fostering supportive leadership and culture significantly enhance access, quality, and efficiency of telemedicine services [32].

Practical equipment for telemedicine scored moderately, with an average of 1.45 out of 3 across specialties. General surgery and pediatrics scored highest, benefiting from standardized and readily available equipment for consultations and follow‐ups, which facilitates implementation. In contrast, internal medicine and neurosurgery require more specialized diagnostic tools and software platforms, which are not uniformly accessible, leading to lower practical readiness. Challenges identified in prior studies include uncertainties in operational mechanisms, insufficient communication and telecommunication infrastructure, and lack of electronic health requirements [34]. Essential telemedicine equipment generally encompasses network and broadcasting devices, IT hardware, software platforms, and medical instruments [35]. Access to these resources, along with adequate bandwidth, significantly influences telemedicine utilization and remains a critical barrier in some specialties [36].

Overall, the evaluation of telemedicine implementation potential across high‐referral specialties demonstrates that readiness depends on multiple factors, including specialists' willingness, organizational capacity, practical equipment, and financial support. Notably, general surgery ranked first overall despite having one of the lowest financial scores. This apparent contradiction can be explained by the specialty's high willingness to adopt telemedicine, strong organizational readiness, and adequate practical infrastructure, which together compensate for financial limitations. Nevertheless, the low financial score highlights potential sustainability risks, underscoring the need for targeted policy interventions such as aligning telemedicine reimbursements with in‐person equivalents or providing dedicated funding to ensure long‐term implementation. Similarly, disparities in other specialties, such as pediatrics, reflect the complex interplay between these dimensions, where lower willingness or practical readiness may limit overall potential despite moderate financial support. These findings emphasize that a holistic assessment of telemedicine readiness must consider all key dimensions rather than focusing on a single factor, guiding both strategic planning and resource allocation for successful adoption.

### 4.1 Limitations

This study demonstrates strengths in its structured methodology, combining expert surveys with quantitative analysis to assess telemedicine feasibility across high‐referral medical specialties. Its evaluation of key indicators—specialists' willingness, financial sustainability, organizational capacity, and practical equipment—provides a comprehensive perspective and ensures informed conclusions, while the use of Likert scales adds objectivity and enhances analytical rigor.

However, the study has several limitations. First, potential subjectivity in expert opinions may limit the accuracy of the findings, as experts' perceptions do not necessarily reflect real‐world operational constraints. In addition, reliance on self‐reported data introduces institutional bias and may overestimate specialists' willingness due to social desirability bias, potentially inflating readiness scores compared with actual clinical behavior. Another limitation was the inclusion of only 31 experts across six specialties. Although the response rate was 100%, the relatively small sample size—reflecting the limited number of eligible experts who possessed both the required clinical expertise and adequate telemedicine‐related experience at Shiraz University of Medical Sciences—may limit the generalizability of the findings. In addition, this study focused solely on the readiness of service providers and organizations and did not examine patients' perspectives or dimensions related to patient readiness, acceptance, user experience, and participation in the implementation of telemedicine; however, these factors are essential for evaluating its acceptability and usability. Another limitation pertains to the geographical and institutional scope of the study. Because the analysis was conducted within a limited set of clinical institutions, findings may not fully represent broader healthcare contexts, such as rural versus urban settings or public versus private hospitals, reducing generalizability and necessitating caution when extrapolating results to the national level. Given that this study was exclusively conducted at Shiraz University of Medical Sciences, the generalization of its findings necessitates careful consideration. Nevertheless, owing to the structural organizational similarities prevalent across many Iranian medical universities, the results may possess a degree of applicability to other medical universities and tertiary referral centers exhibiting analogous referral patterns. However, disparities in infrastructure and resource availability could potentially influence the extent of telemedicine implementation. Finally, the lack of longitudinal follow‐up limits the ability to assess how telemedicine readiness evolves over time, particularly as policy reforms, infrastructure upgrades, and workforce training initiatives emerge. Incorporating longitudinal tracking in future evaluations would therefore provide deeper insights into the stability and sustainability of telemedicine adoption.

Given these limitations, several directions for future research are recommended to strengthen the evidence base for telemedicine implementation. Future research should extend these findings by assessing the long‐term impact and economic sustainability of telemedicine across different specialties. The most immediate and high‐priority direction is conducting mixed‐methods studies that integrate both provider and patient perspectives, addressing the current provider‐centric focus. Further research should also examine the cost‐effectiveness of telemedicine models—particularly in specialties facing financial barriers—and compare implementation across diverse healthcare settings to enhance generalizability. In addition, exploring the integration of artificial intelligence and machine‐learning tools, including pilot testing specialty‐specific decision‐support systems, may offer valuable insights into optimizing telemedicine workflows and improving service quality. Additionally, future studies are recommended to systematically examine factors such as patient experience, digital literacy levels, and access barriers to provide a more comprehensive understanding of the determinants influencing the acceptance and implementation of telemedicine.

### 4.2 Recommendations for Policy and Practice

To translate these findings into actionable strategies, several policy and practice recommendations can be proposed:

Pediatrics: Prioritize reimbursement reforms for pediatric teleconsultations and develop specialty‐specific training modules to address provider concerns and workflow misalignment.

General Surgery: Provide financial incentives and dedicated funding to support acquisition of telemedicine equipment and enhance operational workflows.

Cardiology: Invest in remote monitoring technologies and strengthen integrated tele‐cardiology systems to leverage existing specialist willingness.

General Internal Medicine: Expand digital literacy and telemedicine‐specific competencies through continuous professional development programs.

Orthopedics: Strengthen tele‐rehabilitation platforms, ensuring availability of hardware and bandwidth needed for remote functional assessments.

Neurosurgery: Support high‐resolution imaging transfer systems and ensure data security capabilities tailored to neurosurgical needs.

These specialty‐specific recommendations can help bridge readiness gaps and mitigate the contradictions identified—particularly cases such as general surgery, were high overall readiness contrasts with relatively weak financial capacity.

## 5 Conclusion

This study identified general surgery, cardiology, and internal medicine as the leading candidates for telemedicine implementation within high‐referral specialties, while orthopedics, neurosurgery, and pediatrics demonstrated more limited readiness due to specialty‐specific constraints. Although cardiology's balanced strengths support rapid scale‐up, major gaps—such as limited financing capacity in general surgery and lower willingness among pediatric providers—underscore the need for targeted, specialty‐aligned policy responses. Effective implementation requires tailored strategies, including infrastructure strengthening and workflow integration in internal medicine and orthopedics, financing support for neurosurgery and general surgery, and focused training modules addressing communication and consent challenges in pediatrics. These findings are most relevant to academic referral centers with similar organizational characteristics and may require contextual adaptation in other healthcare settings. Future research should prioritize longitudinal evaluations of telemedicine implementation across specialties, assess patient satisfaction and clinical outcomes alongside provider readiness, and examine the cost‐effectiveness of telemedicine models to guide sustainable adoption. The integration of AI‐enabled decision‐support tools also represents a promising avenue for enhancing the efficiency and accuracy of remote care. Overall, this study provides an evidence‐based foundation for developing realistic, specialty‐specific telemedicine strategies aligned with the operational and financial realities of each discipline.

## Author Contributions

**Reyhane Izadi:** writing – review and editing, investigation, data curation, conceptualization, project administration, methodology. **Abdosaleh Jafari:** conceptualization, project administration, writing – review and editing, validation. **Zahra Zare:** conceptualization, validation, writing – review and editing, project administration. **Mohammad Amin Bahrami:** conceptualization, project administration, writing – review and editing, validation.

## Funding

The authors have nothing to report.

## Ethics Statement

This study complies with the ethical standards of the National Ethics Committee in Iranian Biomedical Research and was approved under code “IR.SUMS.NUMIMG.REC.1402.044” on July 15, 2023.

## Consent

All participants provided written informed consent via a single written question before the survey was conducted and were free to withdraw from the study at any time.

## Conflicts of Interest

The authors declare no conflicts of interest.

## 1 Transparency Statement

The lead author Reyhane Izadi affirms that this manuscript is an honest, accurate, and transparent account of the study being reported; that no important aspects of the study have been omitted; and that any discrepancies from the study as planned (and, if relevant, registered) have been explained.

## Supporting information


Supporting File

## Data Availability

The data that support the findings of this study are available from the corresponding author upon reasonable request.
