# Supplementary material for: Assessing Telemedicine Implementation Potential in High‐Referral Specialties: A Cross‐Sectional Study in Iran
Source: Health Sci Rep. 2026 Aug 26;9(9):e73021. doi: 10.1002/hsr2.73021 (PMC13508582; doi:10.1002/hsr2.73021)
Supplement: Supplementary file 1 [file HSR2-9-e73021-s001.docx]

**Telemedicine Questionnaire**

**Research Title:** Assessing Telemedicine Implementation Potential in High-Referral Specialties

**Purpose:** This questionnaire is designed to gather insights from medical experts at Shiraz University of Medical Sciences regarding the financial, organizational, practical, and workflow aspects of implementing telemedicine in priority specialties. Your responses will remain confidential and will greatly assist researchers and decision-makers in this field.

**Section 1: Respondent Information**

**Expert Category** (Please check one)

☐ Manager / Decision-maker (University/Hospital)

☐ Technical Expert

☐ Medical Expert

**Specialty Area** (Please specify the area you are responding for)

☐ Neurosurgery ☐ Cardiology ☐ General Surgery ☐ Internal Medicine ☐ Orthopedics ☐ Pediatrics

**Section 2: Questionnaire**

| **Dimension** | **Row** | **Question** | **Answer Options** | **Description (Optional)** |
| --- | --- | --- | --- | --- |
| **Financial Security** | 1 | Is there a **dedicated budget** for telemedicine services in your specialty? | ☐ Yes, a dedicated budget exists. ☐ Partial funding is available (but not exclusively for telemedicine in this specialty). ☐ No, there is no dedicated budget. | *Please provide any relevant details:* |
|  | 2 | To what extent is the **current budget** sufficient to support telemedicine services in your specialty? | ☐ Sufficient. ☐ Moderately sufficient. ☐ Insufficient. | *Please elaborate:* |
|  | 3 | Is there a **financial sustainability plan** for telemedicine services in your specialty? | ☐ Yes, a comprehensive plan exists. ☐ A plan exists but requires improvement. ☐ No, there is no plan. | *Please elaborate:* |
|  | 4 | How important is **financial support** in your decision to utilize telemedicine services in your specialty? | ☐ Very important – a key factor. ☐ Somewhat important, but not the only key factor. ☐ Not important – other factors are more critical. | *Please elaborate:* |
| **Organizational Capacity** | 5 | Overall, how much does Shiraz University of Medical Sciences **support** telemedicine in your specialty? | ☐ Strong support. ☐ Moderate support. ☐ Minimal or no support. | *Please elaborate:* |
|  | 6 | Are there **adequate training and resources** for the effective use of telemedicine in your specialty? | ☐ Yes, completely adequate. ☐ Adequate to a certain extent. ☐ No, they are insufficient. | *Please elaborate:* |
|  | 7 | How do you foresee telemedicine impacting daily **workflow efficiency**? | ☐ Improves efficiency (reduces time and effort to complete tasks). ☐ No noticeable change. ☐ Reduces efficiency (increases time or effort to complete tasks). | *Please elaborate:* |
|  | 8 | How do you foresee telemedicine impacting **service quality**? | ☐ Improves service quality. ☐ No noticeable change. ☐ Reduces service quality. | *Please elaborate:* |
|  | 9 | How do you foresee telemedicine impacting **patient/service recipient satisfaction**? | ☐ Increases satisfaction. ☐ No noticeable change. ☐ Decreases satisfaction. | *Please elaborate:* |
| **Practical Requirements** | 10 | Is the **necessary telemedicine equipment** available for your specialty? | ☐ Yes, the main equipment is available. ☐ Available to a limited extent. ☐ No, the main equipment is not available. | *Please elaborate:* |
|  | 11 | Is the telemedicine equipment **regularly maintained, supported, and updated**? | ☐ Yes, regularly. ☐ Occasionally. ☐ Rarely or never. | *Please elaborate:* |
|  | 12 | Is there a **dedicated, suitable space** (considering privacy, lighting, soundproofing, etc.) for conducting telemedicine sessions? | ☐ Yes, fully suitable space is available. ☐ A partially suitable space is available. ☐ No suitable space is available. | *Please elaborate:* |
|  | 13 | Is there a **reliable internet connection** for conducting telemedicine sessions? | ☐ Yes, the connection is highly reliable. ☐ The connection is somewhat reliable. ☐ No, the connection is unreliable. | *Please elaborate:* |
|  | 14 | Are **technical support services** available to address issues during telemedicine sessions? | ☐ Yes, comprehensive support is available. ☐ Limited support is available. ☐ No, technical support is rarely or never available. | *Please elaborate:* |
| **Specialists' Willingness** | 15 | **Specialty:** *What is your medical specialty?* ☐ Neurosurgery ☐ Cardiology ☐ General Surgery ☐ Internal Medicine ☐ Orthopedics ☐ Pediatrics | | |
|  | 16 | **Personal Willingness to provide services via telemedicine** *(Please indicate your personal willingness, regardless of organizational or practical constraints)* ☐ Very Low ☐ Low ☐ Moderate ☐ High ☐ Very High | | |
